# Isotopic measurements of carbon dioxide: the role of measurement science and standards

**DOI:** 10.1007/s00216-023-05000-2

**Published:** 2023-11-17

**Authors:** Juris Meija

**Affiliations:** National Research Council Canada, Ottawa, Canada

**Keywords:** Metrology, Climate change, Carbon isotopes, Air/gases, Geochemistry/geology, Mass spectrometry/IRMS

## Abstract

Isotopic measurements provide valuable information about the origin of greenhouse gases — as carbon dioxide levels increase, there is a corresponding shift towards lighter isotopic composition similar to that of fossil fuels. Detecting such isotopic shifts, however, requires extremely precise measurements, which must also be globally reproducible in order to make reliable policy decisions. This feature article outlines the collective search for the ideal standard for carbon isotope measurements since the 1950s. This tragicomedy of errors, if you wish, has strengthened the reliability of today’s measurements and has taken us from fictional oceans, to toilet seat marbles, and complex mathematical conventions that separate data from reliable results.

## Prologue

In this brief history of carbon dioxide measurement standards, we will sail through imaginary oceans and fly past volcanoes, and along the way we will rediscover ancient squids, and encounter toilet seats, intractable math equations, unstable lithium salts, Kings, and even Michelangelo’s David.

Our interest in isotopic measurements of $\text{CO}_{2}$ starts with the Keeling curve, named after the American scientist Charles David Keeling (1928–2005) whose efforts have led to a meticulous documentation of the steadily rising $\text{CO}_{2}$ levels at the Mauna Loa observatory [1]. Today, the Keeling curve is as iconic to science as Darwin’s finches and the DNA double helix [2].

**Fig. 1:**
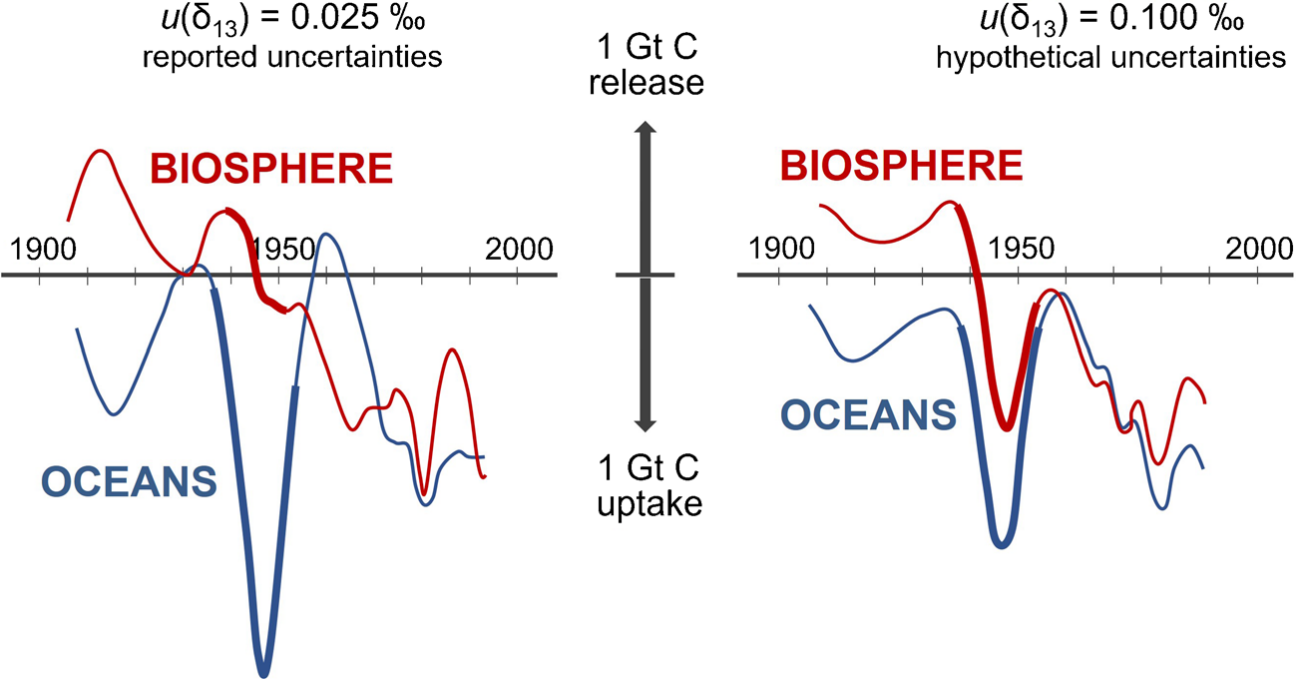
Estimations of global carbon fluxes from biosphere and oceans, by deconvolution of $\text{CO}_{2}$ concentration measurements and carbon isotope measurements, are significantly affected by the quality of carbon isotope measurements [8, 9]. If carbon isotope delta measurements are taken at their face value (that is, having measurement uncertainties of 0.025‰), one concludes that oceans were the major reason for the temporary stabilization of the atmospheric $\text{CO}_{2}$ levels during the 1940s. However, this conclusion becomes much less certain as carbon isotope delta measurement uncertainties increase

The Keeling curve did not just establish the persistent increase in atmospheric $\text{CO}_{2}$ levels, it also showed that the $\text{CO}_{2}$ levels undergo seasonal variations, with plants reducing the amount of $\text{CO}_{2}$ each summer [3]. However, one thing that the Keeling curve does not tell us is where does all the $\text{CO}_{2}$ come from? To answer this question, we must look at the small changes in the isotopic composition of $\text{CO}_{2}$. Because the isotopic composition of carbon is slightly different between the various materials on Earth [4], we can tell if the $\text{CO}_{2}$ is coming from plants, from oceans, or from burning fossil fuels.

Looking at the rising $\text{CO}_{2}$ levels together with radioactive carbon measurements, we observe a shift towards the isotopic signature belonging to fossil fuels (which contain carbon but no ${}^{14}$C [5]) — so that is where most of the $\text{CO}_{2}$ keeps coming from [6]. Overall, carbon isotope measurements of atmospheric $\text{CO}_{2}$ are used to divide the total $\text{CO}_{2}$ levels into components representing contributions from fossil fuels, oceans, and lands, which gives us a more complete picture of the $\text{CO}_{2}$ cycle [7].

There are more nuanced things that isotopes can tell us about the $\text{CO}_{2}$. For example, the Law Dome ice core data show a temporary stabilization of atmospheric $\text{CO}_{2}$ levels during the 1940s. While lots of mathematical modeling has been done to understand this observation [8], taking carbon isotope data at their face value, the models suggest that it was the oceans that absorbed most of the $\text{CO}_{2}$ during the 1940s (Fig. 1). However, Bastos et al. note that “measurement errors in even the best ice-core data currently available make it difficult to accurately quantify variations in the oceanic and terrestrial sinks” [9]. Indeed, we know that the reported carbon isotope ratio measurement uncertainties cannot be fully trusted at their face value: inter-laboratory comparison results often disagree by an order of magnitude more than the reported uncertainties would suggest [10]. In this vein, simulations by Trudinger et al. (Fig. 1) illustrate the importance and the need for high-precision measurements of carbon isotopes in climate studies.

## Act 1: The squid and the toilet seat

Isotopic composition of elements carries an incredible amount of information. For example, by analyzing the oxygen and strontium isotopes in human bones, we can tell that, at a young age, King Richard III moved west (strontium tells us that) to a location where it rained more (oxygen isotopes tell us that) [11]. Similarly, in the 1950s, Harold C. Urey’s team analyzed the changes in oxygen isotopic composition across a small fossil of a Jurassic Squid from North Carolina’s PeeDee region. Urey goes to say that “this Jurassic belemnite records three summers and four winters after its youth, [...], warmer water in its youth than in its old age [and] death in the spring [at] an age of about four years” [12]. This remarkable information about a King who lived 500 years ago or a squid who lived more than 100 million years ago could be gleaned from analyzing the isotopic composition of elements.

These measurements were, and remain to be, comparative measurements whereby one simply seeks to find out the relative differences in the $$ {}^{13}$$C/$${}^{12}$$C isotope ratio of a sample ($$R_\text {sample}$$) and a standard ($$R_{\text{standard}}$$) — a concept that was introduced by Urey in the early 1950s [12]. The resulting quantity is called isotope delta [13]:

1 $$\begin{aligned} \delta ( {}^{13}\text{C}) = R_{\text{sample}}/R_{\text {standard}} - 1 \end{aligned}$$

Carbon isotope measurements took off in the 1950s with five main research groups each using their own standard for comparative measurements at that time [14]. Wickman’s group (Sweden), for example, chose a commercial barium carbonate sample as their standard, whereas others used marble or limestone samples. Still, all chose solid carbonates as their standards, not gases, as solid materials are easier to store and maintain in the long term.

In the end, of course, the reference material of a Nobel laureate was adopted and Urey’s PeeDee belemnite (PDB), made of the family of calcified remains of Jurassic squids described earlier, became the first international standard of carbon isotope measurements.

By the 1960s, however, PDB was all used up and another reference material had to be produced as its replacement [15]. The new material was identified in the early 1980s by an international agreement. This material was far less exotic than a Jurassic squid — it was a crushed marble slab, probably a kitchen counter top, then formally known as the “TS limestone, NBS #19” [16].

This reference material became the new international standard for carbon isotopes for some three decades until it became unavailable to the wider public in mid 2010s. One of the most common questions about this standard is the meaning of the acronym TS. Well, it stands for “toilet seat.” The story goes, it was so named because the color of this material resembled the toilet seats in the Denver Federal Building where this material was established.

The introduction of NBS19 did not change the numerical values of carbon isotope delta measurements, the comparisons were just made to NBS19 from then on instead of to PDB. In order to distinguish between the two approaches, the new scale was called the Vienna PDB (VPDB), because that is where it was adopted [17]. This is similar to other conventions such as the Treaty of Versailles or the Kyoto Protocol which celebrate the places of their adoption.

With more laboratories regularly performing $\text{CO}_{2}$ measurements by the turn of the 21st century, David Keeling and his coworkers lamented that “there are presently no available international isotopic carbonate standards for interlaboratory calibration of $\text{CO}_{2}$” [18]. More reference materials needed to be made.

## Act 2: Nightmare measurements and math

In order to measure carbonate reference materials, they must be first converted to $\text{CO}_{2}$ which can be done by thermal decomposition or by reaction with acids. But carbonates have three oxygen atoms, whereas $\text{CO}_{2}$ has only two; meaning that one oxygen atom goes away which, in turn, leads to isotopic fractionation that depends on the material and the reaction temperature [19], and even the technique employed [20]. Thus, precision measurements require these effects to be taken into account. Yet, the widely accepted value for the ${}^{18}$O/${}^{16}$O isotope fractionation factor, $\alpha (\text {calcite, CO}_{2}) = 1.010\,25$ at 25 ${}^\circ$C, comes from the 1965 measurements (1.010 08 [21]), revised in 1977 based on personal communication [22], despite newer measurements available. It goes without saying that more experimental work is needed to underpin these fundamental factors that affect the measurement results.

Most mass spectrometry measurements pertain to $\text{CO}_{2}$ isotopologues with nominal mass resolution, with the following relationships between the isotope ratios among the isotopologues and the corresponding isotope ratios of carbon and oxygen [23]:

2 $$\begin{aligned} R_{45/44}&= R_{13/12} + 2 R_{17/16} \end{aligned}$$

3 $$\begin{aligned} R_{46/44}&= 2 R_{18/16} + 2 R_{13/12} R_{17/16} + R^{2}_{17/16} \end{aligned}$$

4 $$\begin{aligned} R_{47/44}&= 2 R_{13/12} R_{18/16} + 2 R_{17/16} R_{18/16} + R _{13/12} R^{2}_{17/16} \end{aligned}$$

Since we are interested in the isotope ratios of carbon and oxygen, these equations need to be turned around, which leads to much more complex expressions that no one wants to deal with. For example, the isotope ratio of carbon is obtained by solving the cubic equation (we drop the denominator isotope labels for brevity) [24]:

5 $$\begin{aligned} 5 R^{3}_{13}- &   3 R_{45} R^{2}_{13} + (4 R_{46} - R^{2}_{45}) R_{13} + (4 R_{45} R_{46} - 8 R_{47} \nonumber \\- &   R^{3}_{45}) = 0 \end{aligned}$$

Most mass spectrometers are not equipped to measure the faint signal of the mass 47 isotopologues and they measure only the signals from masses 44, 45, and 46. This means we are short of one equation in order to convert $R_{45/44}$ and $R_{46/44}$ into $R_{13/12}$, $R_{17/16}$, and $R_{18/16}$.

Turns out that the changes in oxygen-17 and oxygen-18 isotope ratios for nearly all terrestrial materials — waters and carbonates alike — fall on a single line, known as the terrestrial fractionation line, whose slope is $\lambda \approx 0.52$ when $\delta ( ^{18}\text {O})$ is plotted against $\delta ( ^{17}\text {O})$ [25]. However, using the terrestrial fractionation line as a shortcut for not measuring isotopologues of mass 47 leads to even more complicated equation [26]:

6 $$\begin{aligned} 3 R^{2}_{13}- &   2 R_{13} R_{45} - 8 (R_{45} - R_{13})^{1/\lambda }/(2K)^{1/\lambda }\nonumber \\- &   R^{2}_{45} + 4 R_{46} = 0 \end{aligned}$$

where $K = R_{17}/(R_{18})^\lambda$. One problem with data reduction, aside the fact that the aforementioned expressions are often solved using linear approximations [26], is to also agree on the values for $\lambda$ and *K*. In the 1950s, the default choice was $\lambda = 0.5$ [14] whereas today we use $\lambda = 0.528$ [26]. And, as noted by Brand et al. [26], this value of $\lambda$ applies only for normal $\text{CO}_{2}$ materials that have been in equilibrium with terrestrial waters. There are, indeed, abnormal materials out there, such as stratospheric $\text{CO}_{2}$, which have widely different values of $\lambda$. For such materials, accurate carbon isotope measurements require independent measurements of their oxygen-17 abundance [23].

**Fig. 2:**
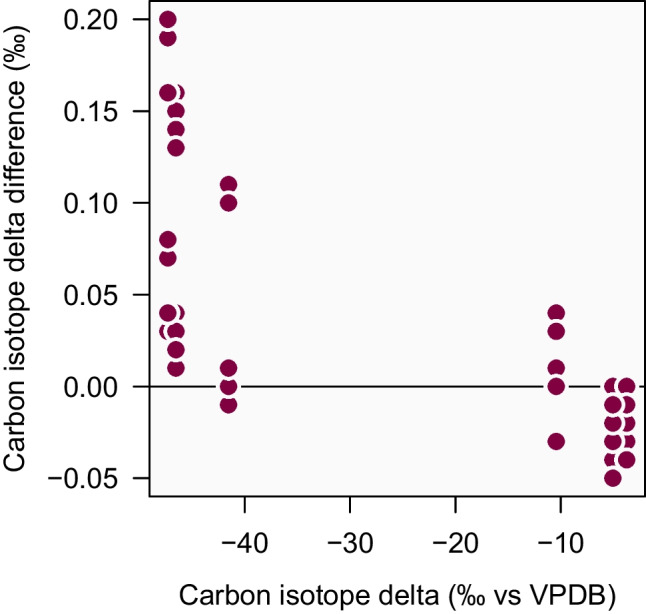
Uncertainty due to data reduction conventions in carbon isotope delta measurements. Inter-laboratory measurements of carbon isotope deltas are reduced using four sets of common assumptions regarding the values of $\lambda$ and *K* (see Eq. 6) [27]. The difference in carbon isotope delta values between these four sets of assumptions reaches 0.2‰

Decisions about the underlying math have a significant effect on the measurement results. In 2004, NIST reported the results of an inter-laboratory study for $\text{CO}_{2}$ measurements [27]. This study involved centralized data reduction and it looked at how much the results would vary depending on what kind of conventional set of assumptions are used for $\lambda$ (and *K*). Four sets of assumptions were investigated, including one that was, at the time, endorsed by the International Atomic Energy Agency (IAEA), and one that is now endorsed by the International Union of Pure Applied Chemistry (IUPAC). As shown in Fig. 2, choosing among these four conventions leads to carbon isotope delta differences of up to 0.2‰. The World Meteorological Organization (WMO) has set data quality objectives for carbon isotope measurements in $\text{CO}_{2}$ aiming at a scale realization compatibility goal of $\pm 0.01$‰ [28]. Hence, a 0.2‰ variation due to mathematical conventions is far from acceptable. And this is one of the reasons why IUPAC has issued recommendations towards unified data reduction algorithms [26] which, in turn, has led to improved inter-laboratory agreement of $\text{CO}_{2}$ isotope measurements [29].

When we actually measure the isotopic composition of carbon in $\text{CO}_{2}$ samples using dual inlet isotope ratio mass spectrometry, the kind of instrumentation used in reference material development [30], there has been a recognized reproducibility problem. A manuscript from 2000 reminds us that inter-laboratory accuracy has hardly improved over the years and that carbon isotope delta measurement results are always showing a familiar pattern: the further the samples are from the reference materials, the bigger the spread between the results [31]. This phenomenon has only been understood some 20 years ago, and it has to do with the cross-contamination.

The way these measurements are done is this: you have a mass spectrometer with two bellows (think accordions), one that is filled with the sample gas, and the other with the reference gas. First we measure the sample gas, and then we switch the changeover valve to the reference gas. At first, the reference gas will be contaminated with small leftovers from the sample gas and the longer one waits before resuming measurements, the smaller the cross-contamination. But one cannot wait forever and, in practice, we inevitably have to deal with small leftovers from the previous gas which leads to slightly biased results. To complicate matters further, no two mass spectrometers are the same, so the magnitude of cross-contamination will differ across the mass spectrometers.

These kinds of considerations are now routinely applied to correct the measurement results, but this was not the case in the past. Indeed, this is one reason why in the 1980s, for example, it was common that carbon isotope delta measurements performed on the same sample by different laboratories might differ by as much as 0.3‰ [32]. In order to improve the coherence of carbon isotope measurements, it was suggested to follow the practice set out with the oxygen scale and adopt a second fixed point [32]. This idea was implemented by the international community two decades later.

A different kind of complication surrounding the measurements of oxygen isotopes lies with the definition of the quantity: the results are reported not for the sample gases themselves but for a hypothetical calcium carbonate sample which would produce identical $\text{CO}_{2}$ gas at $25^{\circ}$C by reaction with concentrated phosphoric acid. Thus, when we do not distinguish between reporting the values for the $\text{CO}_{2}$ gases themselves or the hypothetical carbonates from which these gases could have been made, confusion will arise. For example, two high-level documents provide the isotopic composition of oxygen in the NIST $\text{CO}_{2}$ reference material RM 8564 as $+0.19$‰ [33] and $-10.09$‰ [34], yet use identical notation ($\delta^{18}\text{O}_{\text{VPDB}}$) as a result of a typo in the latter document.

## Act 3: A tragedy strikes

In hopes to improve the agreement between the laboratory results of carbon isotope delta measurements, a second fixed point — a lithium carbonate standard (LSVEC) — was introduced by the IUPAC in 2005 [35]. This followed the same approach taken to improve the oxygen isotope delta measurements in the 1970s when the oxygen isotope delta scale was redefined with two water reference materials (VSMOW and SLAP) as fixed points [36]. One of these fixed points is called the Vienna Standard Mean Ocean Water (VSMOW) despite the fact that Vienna does not have a nearby ocean and that this water is not even an ocean water. The approach of adopting multiple fixed points to define isotope delta scales is similar to how the international Temperature Scale is set up.

**Fig. 3:**
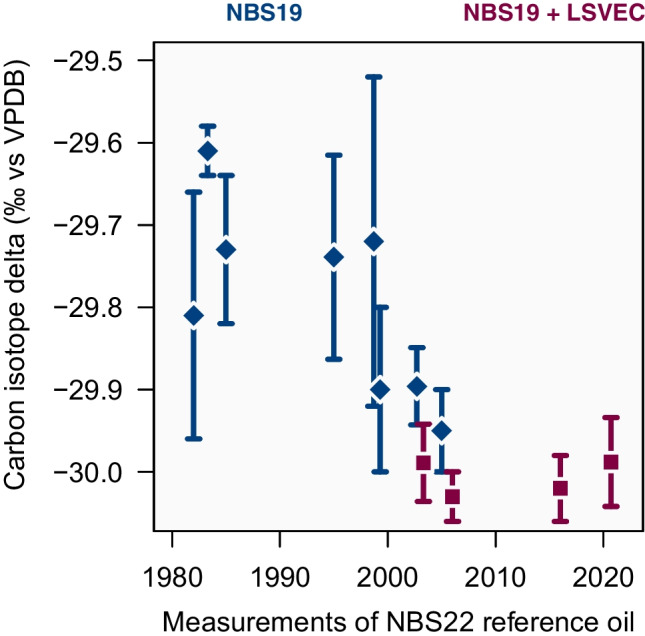
The reference oil NBS22 has been available for more than half a century and its carbon isotope delta measurements provide a snapshot of long-term variability of these measurements

The NBS22 oil is one of the oldest carbon isotope reference materials which has been available since the 1960s [37], and can be used as a quality indicator of carbon isotope delta measurements over time (Fig. 3). When we look at the measurements of this material over the years, we see that the variability in the results has indeed improved since the introduction of the two-point definition of the VPDB scale in 2006. But, we also observe a persistent shift towards more negative isotope delta values.

**Fig. 4:**
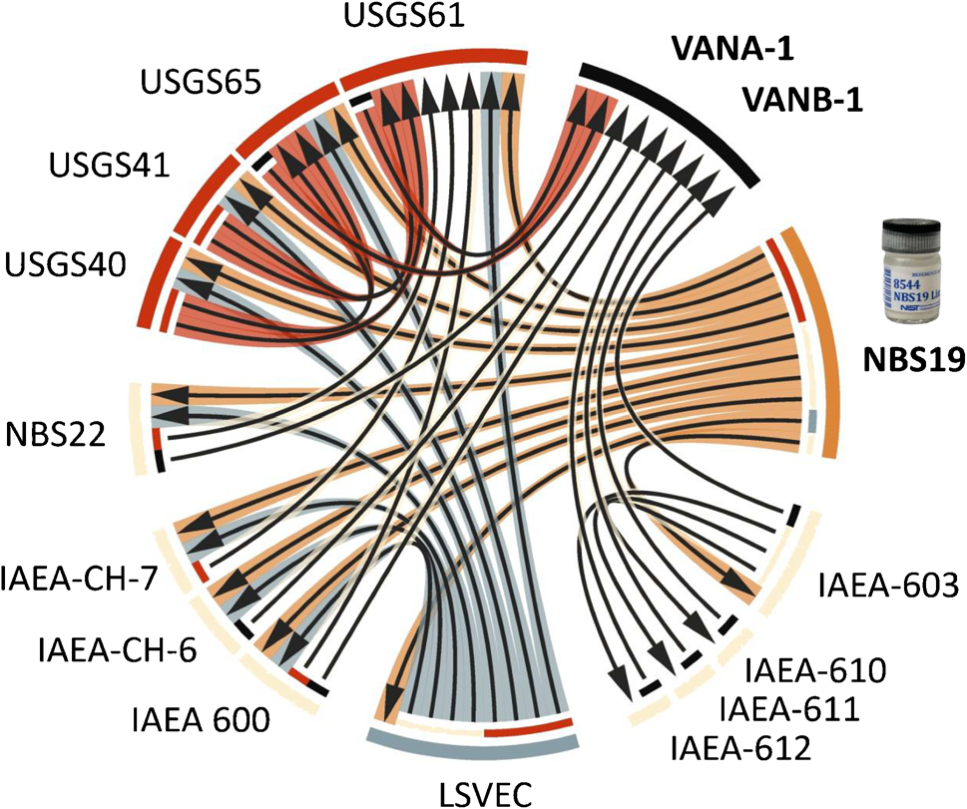
Inter-relationships between carbon isotope reference materials exemplified through a recent certified reference materials of vanillin [39] whose values depend, directly or otherwise, on fourteen other reference materials

A feature of multiple measurement standards is that they are all related somehow [38]. As an example, not long ago NRC Canada produced a reference material of vanillin whose certified isotopic values relied, directly or otherwise, on fourteen other reference materials [39]. And, as shown in Fig. 4, this relationship is not a simple direct chain of comparisons from one standard to another. The big question, of course, is what happens when we find problems with some of these materials.

This happened in 2015 when LSVEC — the same material that was introduced to improve carbon isotope delta measurements — was found to be able to interact with carbon dioxide from air which, in turn, changes the isotopic composition of carbon in this material over time [40]. This was quite a tragedy for the community and can be compared to finding out that the international kilogram prototype is rusting! We can, of course, stop using this material, as IUPAC recommended in 2017 [41], but because many other reference materials have been characterized using LSVEC, as exemplified in Fig. 3, the values assigned to most other carbon isotope reference materials on the market need to be revised if they are to be reported relative to the original VPDB definition set by NBS19 alone.

## Act 4: Michelangelo’s David and the two VPDB scales

These are some of the many reasons that prompted the IAEA to produce a set of new-generation reference materials that would be free of all kinds of measurement issues that have plagued the earlier measurements. The first material of this suite, IAEA-603, was made by grinding up a marble from Tuscany’s Carrara region which is where the marble for Michelangelo’s David also came from [42]. The other three materials, IAEA-610, IAEA-611, and IAEA-612, were commercial calcites [43] and, together, they cover a wide range of carbon isotope delta values. Thus, we now have a suite of high-quality modern reference materials which were prepared in compliance with the ISO Guide 35 on the production of reference materials.

However, only IAEA-603 is certified for both carbon and oxygen isotopes, while the other three materials are certified only for carbon isotopes, and providing reliable values for oxygen isotopes remains to be done, likely by the National Metrology Institutes.

**Fig. 5:**
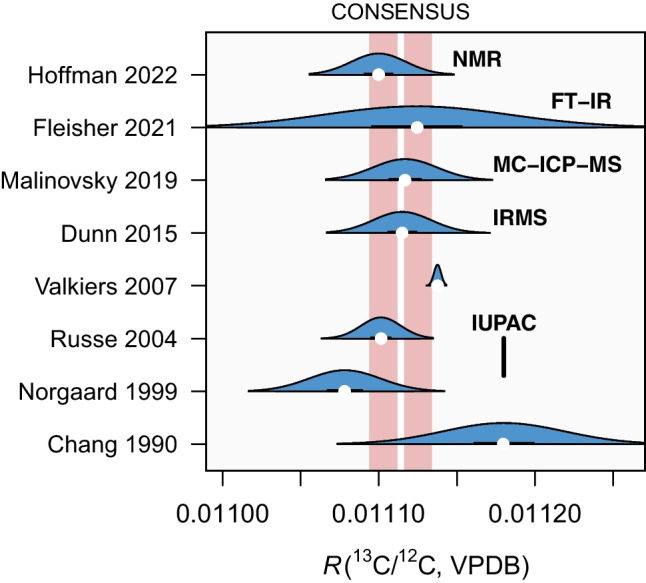
Recent measurement efforts to characterize the isotopic composition of carbon in the Vienna PeeDee Belemnite (VPDB). The consensus value $R_{13,\text {VPDB}} = 0.011\,114$ and its standard uncertainty $u(R_{13,\text {VPDB}}) = 0.000\,010$, calculated by combining the reported results using Bayesian random effects model [51], is significantly lower than the value currently endorsed by the IUPAC [26] by 6‰

Despite the great effort in producing these new calcite reference materials, they don’t quite agree with other reference materials available on the market [44]. When 20+ carbon isotope reference materials are measured against the suite of IAEA calcites, one observes the familiar pattern that Meijer et al. have noted before [31]: the further we are from the VPDB, the bigger the bias between the observed and certified values of these reference materials, reaching 0.2‰ difference for materials whose isotopic composition of carbon is similar to LSVEC [44]. Thus, despite the fact that we have a set of new carbonate reference materials with low associated measurement uncertainties, we are still facing difficulties in connecting measurements that rely on them with the legacy measurements made with older reference materials still available on the market. The recommended values of most reference materials, however, can be made consistent with the IAEA calcites through a one-time adjustment, as suggested by Helie et al. [44].

Part of the problem here is that we rely on materials as references whereas we ought to rely on the well-characterized isotope ratios of elements in these materials as the true reference points [45]. Materials, as we learned from the LSVEC affair, can deteriorate over time, or can be inhomogeneous to begin with.

For several years now, the IAEA-603 calcite is the de facto international standard for all carbon isotope measurements, whereas NBS19 still remains the official scale-defining material. Turns out that assigning a carbon isotope delta value to IAEA-603 relative to NBS19 involves more than just comparing these two materials against one another. In fact, this involves numerous quantities whose values need to be determined one way or another [42]. Because of this, there is “an urgent need” to establish isotope ratio values for all these reference materials so that they can stand on their own [31]. Establishing “absolute” isotopic composition values for our reference materials calls, in essence, for an end of artefacts. The International System of Units went through such a transformation just a few years ago, where seven base measurement units were redefined in terms of fundamental physical constants [46]. One day, perhaps, our isotope scales too will be set by physical constants and not materials.

In line with such aspirations, great efforts have been made to characterize the isotopic composition of the VPDB (Fig. 5). While the measurement uncertainty of $R_{13/12}$ of the VPDB has not significantly improved over the last few decades, our confidence about the isotopic composition of the zero-point on the carbon isotope scale has improved considerably because of the number of independent measurement techniques all providing identical results. Instead of relying on mass spectrometry alone, we have measurements from NMR [47], infrared absorption spectroscopy [48], elemental mass spectrometry (ICP-MS) [49], and molecular mass spectrometry (IRMS) [50], all giving the same result for the ${}^{13}$C/${}^{12}$C isotope ratio of the VPDB.

This paradigm shift would mirror the recent redefinition of the mole: the old definition stipulated that a mole contained as many elementary entities as there are in 12 g of carbon-12. The same goes for the VPDB which is currently defined by the isotopic composition of NBS19 without knowing what it is exactly. Turning both definitions around, the new definition of the mole is now based on the explicit number of elementary entities [52], and the same could be done with the new definition of the VPDB once the measurement science is mature enough for it.

**Fig. 6:**
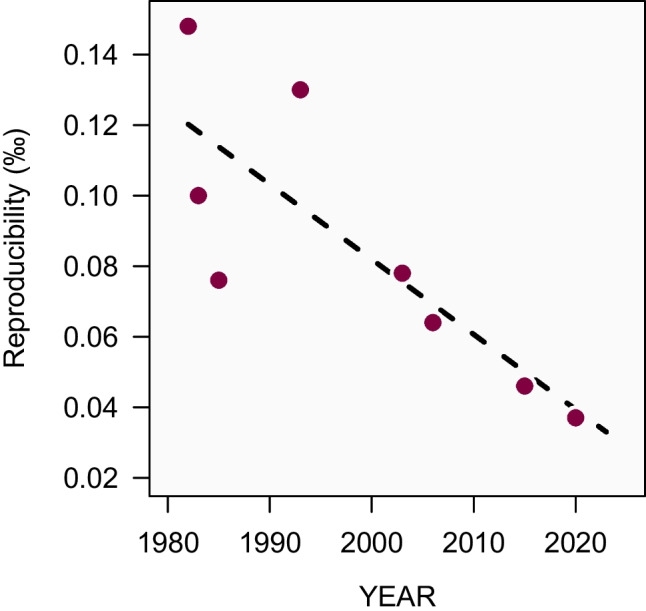
The reproducibility of carbon isotope delta measurement results from major inter-laboratory comparisons is steadily decreasing as a result of sustained metrology research and development efforts. The graph shows robust standard deviations of the reported inter-laboratory measurements of carbonates and $\text{CO}_{2}$ gases (interpolated to a material similar to NBS22) from the following eight studies (with the estimated date of measurements): Schoell-1982 [37], Gonfiantini-1983 [53], Hut-1985 [54], Stichler-1993 [55], Verkouteren-2003 [27], Coplen-2006 [10], Schimmelmann-2015 [56], and Chartrand-2020 [57]

## Epilogue

The collective journey towards making reliable carbon isotope measurements started in the 1950s with the adoption of the first international reference material. Spearheaded by the IAEA, the 1970s saw a new approach whereby these standards were now produced by international collaborations within the scientific community. At the same time, the idea of using multiple fixed points was established to improve oxygen isotope delta measurements, and this was also later extended to carbon. And just recently we have witnessed a new-generation of high-quality reference materials from the IAEA that comply with the ISO Guide 35 on the production of reference materials. These incremental advances have all contributed to a steady progress towards more reliable carbon isotope measurements (Fig. 6), and has brought the measurement science within the reach of the very tough WMO data quality objectives.
